# Noisy Galvanic Vestibular Stimulation Sustainably Improves Posture in Bilateral Vestibulopathy

**DOI:** 10.3389/fneur.2018.00900

**Published:** 2018-10-22

**Authors:** Chisato Fujimoto, Naoya Egami, Takuya Kawahara, Yukari Uemura, Yoshiharu Yamamoto, Tatsuya Yamasoba, Shinichi Iwasaki

**Affiliations:** ^1^Department of Otolaryngology and Head and Neck Surgery, Graduate School of Medicine, University of Tokyo, Tokyo, Japan; ^2^Biostatistics Division, Clinical Research Support Center, University of Tokyo Hospital, Tokyo, Japan; ^3^Educational Physiology Laboratory, Graduate School of Education, University of Tokyo, Tokyo, Japan

**Keywords:** bilateral vestibulopathy, galvanic vestibular stimulation, electric stimulation, posture, vestibular diseases

## Abstract

Patients with bilateral vestibulopathy (BV) suffer from persistent postural imbalance, leading to a marked decrease in quality of life and a higher risk of falls. However, so far, the effective treatments for BV are very limited. We examined whether long-term noisy galvanic vestibular stimulation (nGVS) keeps improving body balance after the cessation of the stimulus in BV patients. Thirteen BV patients received nGVS for 30 min with a lower intensity than the intensity at which they feel any cutaneous sensations, and their postural movement was monitored for 6 h after the stimuli. The same session was repeated at 14-day intervals. Stance tasks on two legs were performed with eyes closed. The velocity of the center of pressure (COP) movement, the area enclosed by the COP movement, and the root mean square of the displacement of the COP were measured. The power spectrum of the COP movement was assessed. Subjective improvement of body balance was graded as worsened (−2), slightly worsened (−1), unchanged (0), slightly improved (+1) and improved (+2) in comparison with that without nGVS. In each session, the velocity of the COP movement was significantly improved for 6 h after the stimulus had ceased (*P* < 0.01). Concomitantly, the mean frequency of the COP power spectrum was significantly reduced in the anterior-posterior axis (*P* < 0.05). Subjective symptoms of imbalance were improved during the post-stimulation effect (*P* < 0.05). nGVS leads to an improvement in body balance that lasts for several hours after the end of the stimulus in BV patients with a reduction in the high-frequency components of their postural movement. This trial was registered with the University Hospital Medical Information Network (UMIN) Clinical Trials Registry (UMINCTR: UMIN000028054).

## Introduction

The vestibular system is a sensory system that serves to stabilize body balance by providing information about the angular and linear accelerations of the head (1). Bilateral involvement of the vestibular end organs and/or the vestibular nerve (bilateral vestibulopathy, BV) causes persistent unsteadiness, particularly in darkness or when moving on uneven ground (2–4), leading to a marked decrease in health-related quality of life indices (5) and a higher risk of falls (6). However, so far, the effective treatments for BV are very limited.

Galvanic vestibular stimulation (GVS) delivers electrical current transcutaneously to the vestibular end organs and their afferents through electrodes placed over the mastoids (7, 8). It has been used to examine the role of vestibular signals in the control of gaze, posture and locomotion (7). Recently, an imperceptible level of GVS delivered as zero-mean current noise (noisy GVS, nGVS) has been reported to facilitate the processing of weak, subthreshold stimuli in neural systems, for example the amelioration of baroreflex function in healthy adults and autonomic and motor functions in patients with neurodegenerative diseases (9–11). We have previously shown that a 30-s application of nGVS can improve postural stability in healthy subjects and in BV patients (12). nGVS also enhances the vestibulo-ocular reflex in healthy subjects (13) and the vestibulo-spinal reflex in healthy subjects and BV patients (14). Moreover, nGVS improves walking stability in healthy subjects and BV patients (15–17). The proposed explanation behind these effects is considered to be stochastic resonance, in which the existence of an optimal level of noise can enhance information processing in a non-linear system (18, 19).

We recently published a novel finding that nGVS also has a post-stimulation effect on the improvement of postural stability in healthy elderly adults (20). The study indicated that a 30-min or 3-h application of nGVS lead to a sustained improvement in postural stability that lasts for several hours without any adverse events, even after the cessation of the stimulus. This newly discovered effect may be caused by neuroplasticity in the brainstem and/or cerebellum induced by nGVS.

In the present study, we examined whether nGVS has a post-stimulation effect on the improvement of body balance in BV patients as observed in healthy adults in our previous study (20). We revealed that imperceptible levels of nGVS can improve body balance in BV patients for several hours after the cessation of the stimulus with a reduction in the high-frequency components of their postural movement. This long-term post-stimulation effect of nGVS may further increase the clinical usefulness of nGVS as a new minimally-invasive approach for untreatable postural instability in the BV.

## Materials and methods

### Standard protocol approvals, registrations, and participant consents

This study was conducted in accordance with the Helsinki Declaration and was approved by the Institutional Review Board of the University of Tokyo Hospital (P2016-011). This trial was registered with the University Hospital Medical Information Network (UMIN) Clinical Trials Registry (UMIN-CTR: UMIN000028054). Written informed consent was obtained from all participants.

### Participants

**Figure 1:**
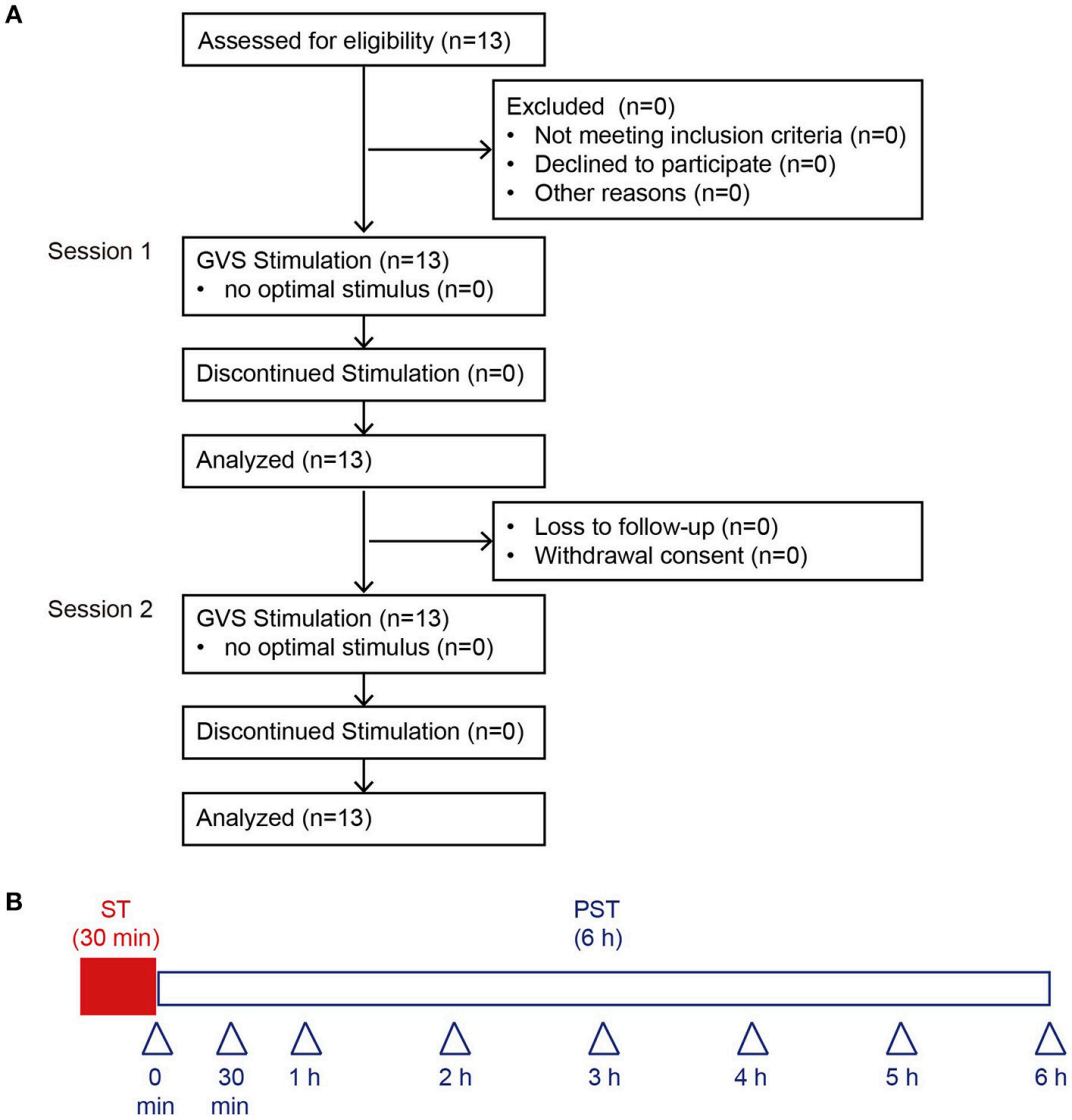
Consolidated Standards of Reporting Trials diagram through the study and protocol. **(A)** We recruited 13 BV patients. An optimal stimulus was obtained before each session from all 13 patients, who were all included in the final analyses. **(B)** This protocol was performed in Session 1 and Session 2. Arrowheads indicate the measurement time point of posturography. BV, bilateral vestibulopathy; ST, stimulation period; PST, post-stimulation period.

Thirteen BV patients (8 men and 5 women; age range 43–83 years, mean age 63.1 [± 4.0] years) were enrolled from the Balance Disorder Clinic, Department of Otolaryngology and Head and Neck Surgery, the University of Tokyo Hospital (Figure 1A). The rationale for this sample size is described in the Data analysis section. The etiologies of vestibulopathy in the 13 patients are shown in Table 1. All the patients showed bilateral catch-up saccades in the horizontal plane during head impulses and showed reduced or absent responses to ice water caloric irrigation of the external auditory canal bilaterally (maximum slow phase velocity of nystagmus <10°/s) (21). Development of otolith function tests such as vestibular evoked myogenic potentials (VEMPs) has led to progress in the pathophysiological investigation of otolith organ dysfunction. Recently, we reported that BV patients with absent VEMPs in the presence of normal caloric responses bilaterally (21, 22). However, originally, BV was diagnosed using tests for horizontal semicircular canal function (2, 4). In the present study, we diagnosed BV according to the conventional method.

**Table 1:** The etiologies of vestibular dysfunction in the enrolled BV patients and their optimal intensities for nGVS.

**Patient**	**Age (year)/Sex**	**Etiology**	**Optimal intensity (**μ**A)**	
			**Session 1**	**Session 2**
1	78/M	Unknown	500	300
2	83/F	Unknown	200	200
3	46/M	Unknown	700	1000
4	54/M	Mitochondrial A3243G mutation	700	300
5	49/M	Unknown (unilateral temporal bone fracture)	700	500
6	72/M	Bilateral vesitibular neuritis	300	300
7	49/F	Unknown (unilateral vestibular neuritis)	200	300
8	80/M	Unknown	300	500
9	54/F	Mitochondrial A3243G mutation	300	700
10	74/F	Unknown	300	700
11	43/F	Unknown	500	200
12	64/M	Unknown	700	500
13	74/M	Unknown	500	500
Mean			454	462
S.E.			55	65

*BV, bilateral vestibulopathy; nGVS, noisy galvanic vestibular stimulation; S.E., standard error*.

### Procedures

nGVS was delivered at its optimal intensity for 30 min (stimulation period, ST) and participants were then monitored without further stimulation for 6 h (post-stimulation period, PST) (Session 1). Postural stability was measured after the nGVS for 30 min (Figure 1B). The duration of posturography measurement at each time point after 30-min application of nGVS was 30 s. Except for the period when postural stability was being measured, participants were allowed to move freely in the hospital. Fourteen days after Session 1, we performed Session 2 to assess the reproducibility of the measured results in Session 1. The procedures for Session 2 were the same as Session 1 (Figure 1B).

### Posturography

Real-time measurements of changes in the position of the center of pressure (COP) were provided by using a Gravicorder GP-5000 (Anima Co. Ltd., Tokyo, Japan; sampling frequency, 20 Hz). Two-legged stance tasks were performed by the participants with and without nGVS in an eyes-closed condition. We did not use a foam rubber in the present study, because some BV patients cannot stand on the foam rubber for 30 s. The following three COP parameters were measured in the XY plane: the mean velocity of the COP movement (velocity), the area enclosed by the COP movement (area), and the root mean square (RMS) of the displacement of COP (12, 20). The power spectrum of the acceleration signal of the COP was estimated using the maximum entropy method with a frequency resolution of 0.001 Hz and a lag value of 120 (23, 24). The power spectral density (PSD) of the COP was calculated for the anterior-posterior (AP) and the medial-lateral (ML) axes between 0 and 10 Hz. Mean frequency (MF) was used as a parameter for the frequency component change.

### nGVS

The procedure of nGVS application has been described previously (20). In brief, white noise GVS, ranging from 0.02 to 10 Hz, was applied with electrodes placed over the right and left mastoids by a portable stimulator. GVS waveforms were digitally stored and converted from digital to analog at 20 Hz. The optimal intensity of nGVS for each participant was determined before starting each session. Initially, the value of each COP parameter without nGVS (0 μA) for 30s was measured and this was defined as the baseline value. Subsequently, nGVS was applied for 30s with peak amplitudes set at 100, 200, 300, 500, 700, and 1,000 μA. The optimal intensity was defined as the intensity at which the value measured during the stimulation was smaller than that at baseline simultaneously in all of the three COP parameters, as previously described (20). If a participant felt any sensations in response to nGVS at a certain intensity, it was not adopted as the optimal intensity.

### Subjective improvement

Subjective improvement of body balance was evaluated after posturography testing at each time point in each session and was graded as worsened (–2), slightly worsened (–1), unchanged (0), slightly improved (+1) and improved (+2) in comparison with that at baseline.

### Outcome measures

In each session, posturographic data were analyzed at 0, 0.5, 1, 2, 3, 4, 5, and 6 h during the PST (Figure 1B). The primary endpoint was defined as the amount of change in velocity from baseline until PST 3 h in Session 1. Our previous study for healthy subjects showed that 30-min nGVS had a post-stimulation effect on the improvement in the velocity for 4 h (20). On the other hand, the post-stimulation effect on the improvement in the area and RMS was 3 h, shorter than that in the velocity. Therefore, we selected the velocity as the primary endpoint. Secondary endpoints were defined as follows: (1) amount of change from baseline until PST 6 h of the velocity, area, and RMS in each session; (2) longitudinal change in subjective improvement in each session; (3) determined optimal intensity in each session. We set determined optimal intensity in each session as one of the secondary endpoints, because we compared the optimal intensity between Session 1 and Session 2.

### Data analysis

Primary endpoint measurements were analyzed using a linear mixed model with outcome change from baseline at each measurement time point as a response value, measurement time points as fixed effects (without intercept term), and each patient as a random effect. An F test for the fixed effect was conducted. In our previous study of 11 BV patients (12), the velocity at the baseline decreased from 104.6 cm (standard deviation 39.5)/30 s to 70.1 cm (standard deviation 27.4)/30 s immediately after the optimal intensity of nGVS. Therefore, we assumed that the effect of the optimal intensity of nGVS on the velocity was about 30 cm/30 s decrease. We assumed a decrease in velocity of 30 cm in 30 s held until PST 3 h in Session 1. To confirm the effect by F test for the fixed effect using the model described above with a two-sided alpha error of 0.05, a power of 90%, a standard deviation of velocity of 50 cm in 30 s, and a correlation of velocity among each measurement time point of 0.7, would require 10 patients. Assuming a 20% dropout, we aimed to recruit 13 patients in this trial. Secondary endpoints (1) and (2) were analyzed using a linear mixed model with the outcome measure at each measurement time point as a response value, measurement time points as fixed effects (without intercept term), and each patient as a random effect. A test for contrast between the baseline and all measurements in PST was conducted to assess the average decline from baseline. A student's *t*-test was also conducted on the change from baseline for each measurement in PST. The optimal intensity in each session (secondary endpoint (3)) was compared using a paired *t*-test. A paired *t*-test was also conducted for the comparison between the absolute value at baseline between Session 1 and Session 2. We defined the normalized ratio (NR) as the ratio of the value of each parameter at the measurement time point to that at baseline, and we illustrated the longitudinal change using NR in the graph. Data are expressed as mean ± standard error. All statistical analysis was performed using SAS software version 9.4 (SAS Inc., Cary, NC, USA). *P* < 0.05 was considered statistically significant.

## Results

### Determining the optimal intensity of nGVS for postural stability in BV patients

Prior to examining the post-stimulation effect of nGVS in BV patients, we determined the optimal intensity of nGVS, for improving the velocity, area and RMS of the COP simultaneously during the 30 s stimulus as compared to the control without nGVS. The optimal intensity of nGVS was obtained for all 13 BV patients; the mean intensity was 454 (± 55) μA in Session 1 and 462 (± 65) μA in Session 2 (Figure 1A, Table 1). There were no significant differences between them [*P* = 0.92 (for paired *t*-test)].

### Post-stimulation effect of nGVS on the improvement in postural stability in BV patients

**Figure 2:**
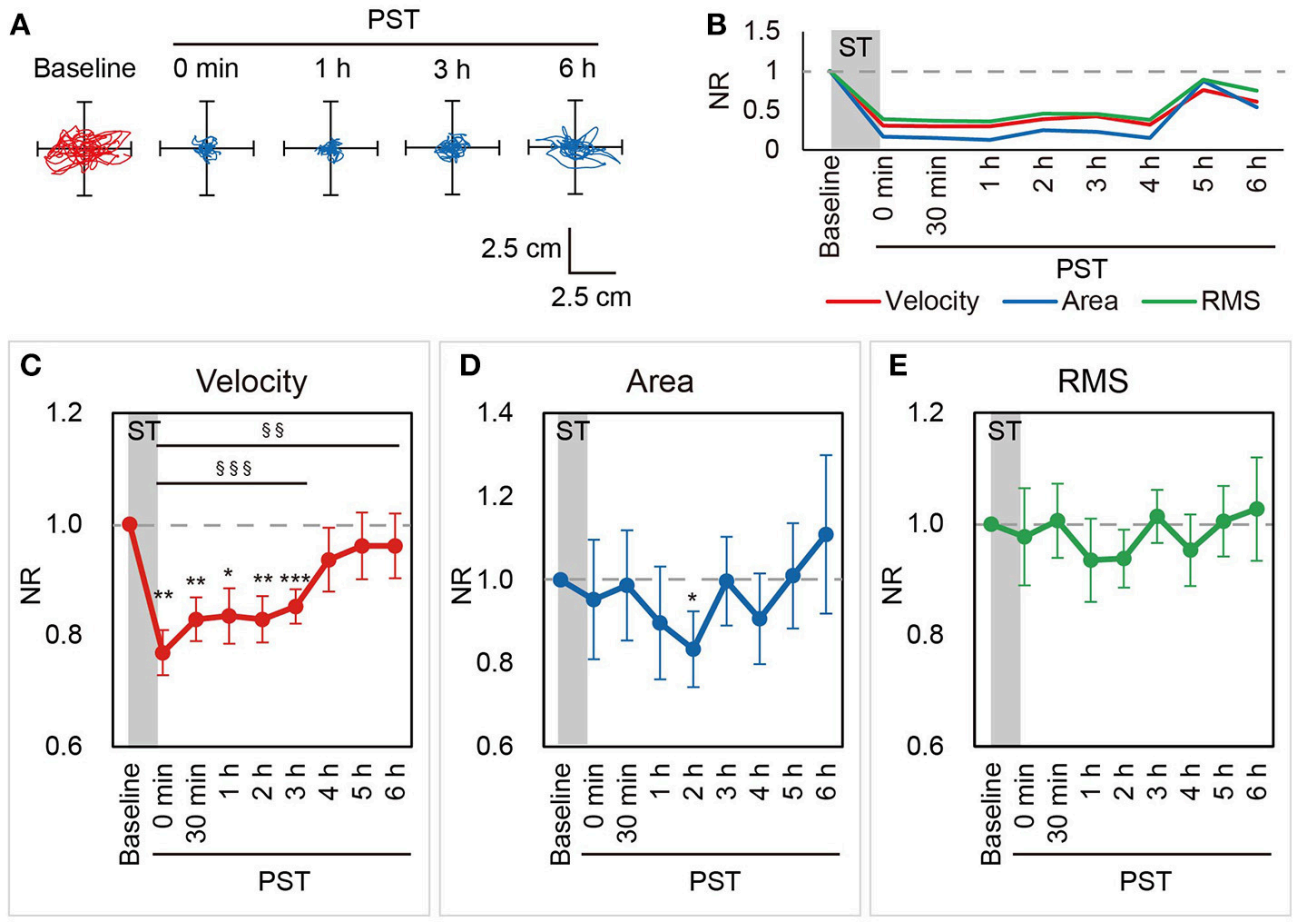
NRs of the velocity, area, and RMS in Session 1. **(A)** Statokinesigrams for a representative BV patient, a 46-year-old male patient (Patient #3), in the first nGVS session (Session 1) are shown. **(B)** Changes in the NRs of the velocity, area, and RMS for Patient #3 in Session 1 are shown. nGVS for 30 min improved the NRs of all three parameters in the PST. **(C–E)** Mean NRs of the velocity **(C)**, area **(D)**, and RMS **(E)** in Session 1 are shown. Dashed line indicates NR = 1.0. NR, normalized ratio; RMS, root mean square; ST, stimulation period; PST, post-stimulation period. ^§§^*P* < 0.01, ^§§§^*P* < 0.001 (for contrast test). ^*^P < 0.05, ^**^P < 0.01, ^***^P < 0.001 (for Student's t-test).

The optimal intensity of nGVS was applied for 30 min and we evaluated the post-stimulation effect of nGVS on postural stability for 6 h after the end of the stimulus in BV patients (Supplementary Figures 1–3). Figures 2A, B show a representative patient with BV, who received nGVS at their optimal intensity of 700 μA for 30 min. The patient showed improvements in the NRs of all three parameters up to 6 h after the cessation of the stimulus.

In Session 1, the NRs of the velocity were smaller at the immediate PST (PST 0 h) than at baseline in all patients (100%) and the mean NR was 0.77 (±0.04). The NR of the velocity was consistently smaller than baseline until PST 3 h in most patients [11 patients (85%) at PST 0.5 h, 12 patients (92%) at PST 1 h, 11 patients (85%) at PST 2 h, 12 patients (92%) at PST 3 h]. Although this ameliorating effect of nGVS tended to decrease thereafter, the mean NR of the velocity was still smaller than at baseline until PST 6 h (Figure 2C). A longitudinal analysis revealed that velocity was significantly smaller until PST 6 h than at baseline [*P* < 0.001 until PST 3 h and *P* = 0.004 until PST 6 h, contrast test; *P* = 0.003 until PST 3 h, *F*-test] (Figure 2C). On the other hand, the longitudinal analysis did not reveal a significant improvement in the area [*P* = 0.127, until PST 6 h, contrast test] or the RMS [*P* = 0.406 until PST 6 h, contrast test]. The ameliorating effect of nGVS on the area was the strongest at PST 2 h, at which there was a significant improvement compared to baseline [10 patients (77%) showed the improvement of NR; *P* = 0.025, Student's *t*-test] (Figure 2D). As for the RMS, while 9 patients (69%) showed an improvement of the NR at PST 2 h, no significant improvement of the NR was confirmed [*P* = 0.142, Student's *t*-test] (Figure 2E).

**Figure 3:**
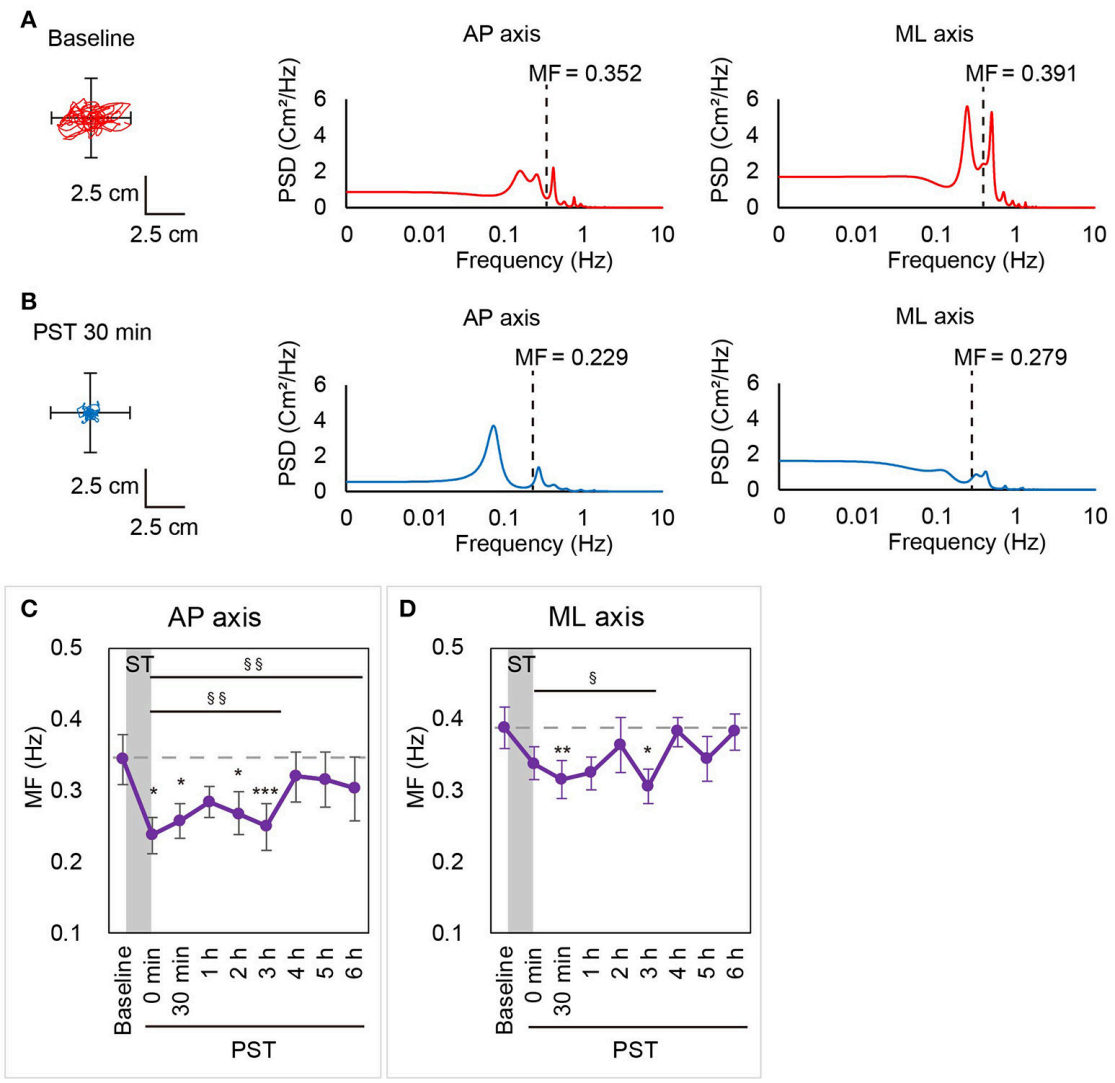
MF calculated by power spectral analysis in Session 1. A statokinesigram and a power spectral density curve for Patient #3 in Session 1 at baseline **(A)** and at PST 30 min **(B)** are shown. MF was lower at PST 30 min than at baseline in both AP and ML axes. **(C,D)** MF in the AP axis **(C)** and the ML axis **(D)** in Session 1 are shown. Dashed line indicates mean MF at baseline. AP, anterior-posterior; ML, medial-lateral; MF, mean frequency; ST, stimulation period; PST, post-stimulation period. ^§^*P* < 0.05, ^§§^*P* < 0.01 (for contrast test). ^*^P < 0.05, ^**^P < 0.01, ^***^P < 0.001 (for Student's t-test).

To obtain further insights into the effect of nGVS on postural stability in BV patients, we then investigated changes in the frequency distribution of the COP movement by power spectral analysis. Figures 3A,B show a power spectral density curve of the COP in the patient shown in Figures 2A,B at the baseline and PST 30 min. In this patient, the frequency distribution of COP movement shifted to lower frequency components at PST 30 min as compared to the baseline in the AP axis as well as in the ML axis. A longitudinal analysis for all the patients revealed that MF was kept lower than at baseline until PST 6 h in the AP axis [*P* = 0.008, contrast test] and until PST 3 h in the ML axis [*P* = 0.018, contrast test] (Figures 3C,D). These results suggest that the reduction in high frequency components of the COP movement by nGVS has a strong association with the improvements in postural stability.

To assess the reproducibility of the results in Session 1, we repeated the same process 14 days later (Session 2). As for the absolute value, the velocity at baseline of Session 1 and Session 2 was 3.40 (±0.36) and 3.42 (±0.41), respectively. There was no significant difference of the velocity at baseline between Session 1 and Session 2 [*P* = 0.904, paired *t*-test]. Similarly, there was no significant differences of the area and the RMS at baseline between Session 1 and Session 2 [*P* = 0.707 for the area, *P* = 0.285 for the RMS, paired *t*-test]. The results in Session 2 were almost the same as in Session 1 (for example, *P* = 0.892, test for interaction of the velocity). The velocity was significantly smaller than at baseline throughout the 6 h PST in the longitudinal analysis [*P* = 0.002, contrast test] whereas no significant improvement was confirmed for the area or RMS throughout the 6 h PST as compared to the baseline in the longitudinal analysis [*P* = 0.236 (area), *P* = 0.210 (RMS), contrast test] (Supplementary Figure 4). The shift of the MF of the power spectrum to a lower frequency compared to baseline was also confirmed in the AP axis [*P* = 0.029 until 6 h, contrast test] (Supplementary Figure 5).

All through Sessions 1 and 2, the only adverse event that might have been associated with the nGVS was a right-sided hearing loss observed in one patient (Patient #3). However, this patient had complained of this symptom before the start of nGVS in Session 1, so it is impossible to ascertain whether there was a causal relationship between the nGVS and this hearing loss.

### Subjective improvement in postural stability after nGVS in BV patients

**Figure 4:**
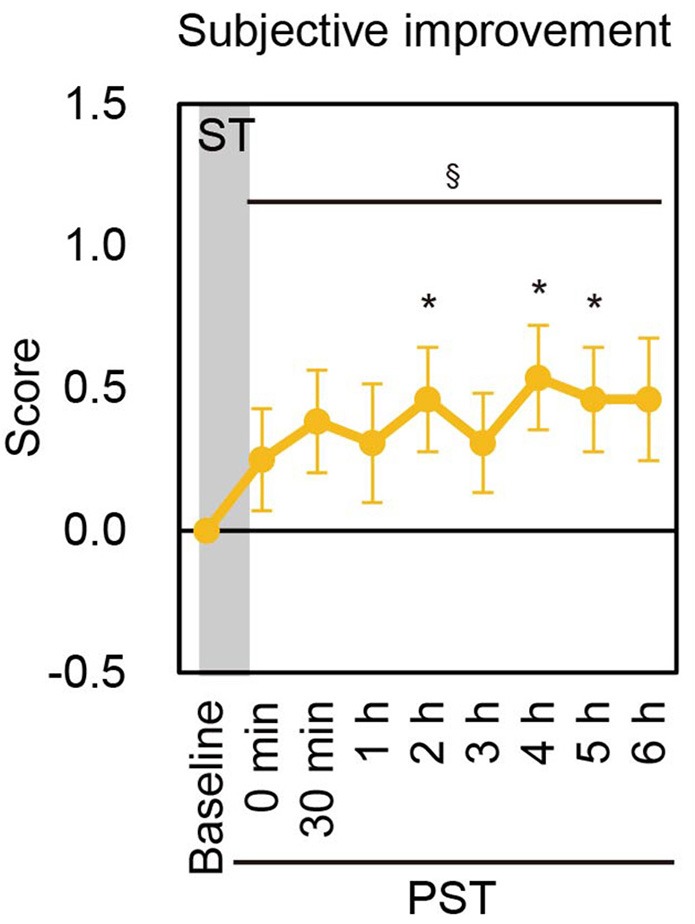
Subjective improvement score in Session 1. Mean score of subjective improvement at baseline and each PST time point in Session 1 are shown. ST, stimulation period; PST, post-stimulation period. ^§^*P* < 0.05 (for contrast test).^*^P < 0.05 (for Student's t-test).

The subjective improvement of postural stability after the cessation of nGVS was assessed by the patients' own rating of their body balance during posturography as compared to baseline. Of the 13 BV patients, seven reported subjective improvements for at least one measurement point in the PST in each session, whereas three patients in Session 1 and one in Session 2 reported subjective worsening for at least one point. The mean score of subjective improvement was consistently higher compared to baseline until PST 6 h in both sessions. A longitudinal analysis revealed that the scores of subjective improvement were significantly improved up to PST 6 h [*P* = 0.033 (Session 1), *P* = 0.020 (Session 2), contrast test] (Figure 4, Supplementary Figure 6).

## Discussion

In the present study, we examined the effects of the long-term application of nGVS on static postural stability in patients with BV. We found that nGVS has a significant post-stimulation effect of postural improvement that lasts for several hours in BV patients, especially in the velocity of the COP movement. Power spectrum analysis showed that nGVS significantly reduced the high frequency component of the COP movement during the post-stimulation period. Furthermore, nGVS also improved subjective body balance during the post-stimulation period. These effects support the applicability of nGVS for improving postural stability in BV patients during the activities of daily life.

BV leads to severe symptoms such as persistent unsteadiness and oscillopsia during head and/or body movements (4), but effective treatments have been very limited so far. A vestibular implant, which electrically stimulates the peripheral vestibular nerves through implanted electrodes, has been proposed as a potential therapy for BV (25–27). However, this treatment has some problems including the risks associated with invasive surgery, which can lead to hearing loss and facial nerve palsy. In contrast, nGVS is minimally invasive and easy-to-use for improving body balance in BV patients. nGVS provides a potentially promising treatment option for BV.

We revealed that nGVS was effective in improving postural stability in BV patients, especially in the velocity of COP movement. BV causes persistent unsteadiness (2–4), leading to a greater risk of falling (6). Previous reports showed that the greater COP velocity and COP sway length were closely associated with the greater fall risk (28, 29). The effect of nGVS on postural improvement may lead to decrease in the risk of falls in BV patients.

The mechanism underlying the ameliorating effect of nGVS on postural stability during the stimulation is thought to be stochastic resonance. An optimal amount of noise intensity has been shown to strengthen the detection of subthreshold inputs in various sensory systems such as tactile sensation, auditory perception and visual perception (30–32). In the present study, we were able to find an optimal intensity of nGVS in all participants, which caused the simultaneous improvement of all three COP parameters during stimulation, consistent with our previous reports (12, 20).

This study and our previous study revealed that nGVS had post-stimulation effects for improving postural stability for BV patients and healthy elderly adults (20). The mechanism underlying this phenomenon might be the neuroplasticity induced by nGVS in the central vestibular system. Synaptic plasticity in the vestibular system involves both the cerebellar circuits and the vestibular nuclei. The cerebellum shows long-term depression at the parallel fiber to Purkinje cell synapses, which underlies the early stages of vestibular learning (33, 34). The vestibular nuclei, which provide an excitatory input to ipsilateral extensor motor neurons of the legs and an inhibitory input to reciprocal flexor motor neurons through the lateral vestibulospinal tract (35), also show long-term potentiation and long-term depression induced by high-frequency vestibular nerve stimulation (36). These neuroplastic changes in the vestibular nucleus are thought to underlie the storage of consolidated memories of the vestibular system (36). The postural improvement in the PST associated with nGVS might be due to the induction of neuroplasticity in the vestibular nucleus and/or the cerebellum.

While the post-stimulation effect of nGVS on postural stability was observed only in the velocity of the COP in BV patients in the present study, nGVS improved all the three parameters of the COP in healthy elderly subjects in our previous study (20). One reason for this difference in the results between the healthy elderly adults and BV patients may be the difference in effectiveness of plasticity induction in the vestibular system between the healthy adults and the BV patients. Since synaptic plasticity in the vestibular nucleus is induced by high frequency stimulation of the primary vestibular afferents (36), it is possible that nGVS-induced plasticity in the vestibular nerve may be smaller in the BV patients than in the healthy adults. Another possible reason is the difference in posturography measurement methods between the previous study and the present study. In the previous study, two-legged stance tasks were performed while standing on a foam rubber surface with eyes closed (20). Under these conditions, the role of vestibular inputs enhanced by nGVS might become more pronounced while visual and somatosensory inputs were reduced, resulting in more pronounced post-stimulation effects on postural stability.

In our previous study, we applied nGVS for 30 min with the optimal intensity in healthy subjects and monitored their postural movement in eye closed/foam rubber condition for several hours after the stimuli (20). This study demonstrated that 10–20% postural improvement in the velocity was shown until 4 h after the stimuli. This improvement of postural stability observed in healthy subjects was almost comparable to that observed in BV patients in the present study. It is possible that, in both the healthy subjects and the BV patients, the post-stimulation effect of 30-min nGVS on the improvement of the velocity could be 10-20% regardless of whether the foam rubber is used. To investigate this possibility, a new study regarding long-term nGVS in both BV patients and healthy controls is necessary.

In the present study, the shift to the lower frequency components of the COP movement was observed during the post-stimulation period. This might be because changes in the high frequency component of the COP are reflected more clearly in the velocity of the COP than they are in the area or RMS of the COP. It has been previously reported that patients with peripheral vestibulopathy showed an increase in the middle to high-frequency components of COP movement while standing on a stable platform (37). Our group also demonstrated that patients with peripheral vestibulopathy showed an increase in activity of middle to high frequency movement (>0.1 Hz) during static posturography (23). Another group showed that elderly BV patients had greater energy consumption of COP at high frequencies (>0.78 Hz) (38). Our result suggests that nGVS can attenuate the high frequency component of COP movement, which is characteristic in patients with vestibulopathy, to bring them closer to the normal range of frequency components of postural sway in healthy subjects. The corrections in the higher frequency component could be representing that the nGVS can stimulate specifically the error signal of the control system adapting these frequencies.

The present study has several limitations. First, this study was a single-arm trial without a placebo control group. This study was designed as a feasibility study to examine the long-term effects of nGVS in patients with bilateral vestibulopathy before conducting a pivotal study, a double-blind placebo-controlled crossover study. It is possible that placebo effects might have affected the subjective improvement score. A confounding bias and a selection bias might also exist. The effects of habituation of the participants due to the repeated measurements of posturography on the values of parameters during the post-stimulation period could not be evaluated, because there was no placebo control group. However, the mean NR of the velocity increased after PST 4 h in each session. These results cannot be explained by the influence of habituation and suggest that the post-stimulation effect of nGVS certainly exists and has been attenuated. The second limitation is that gender differences could not be assessed due to the small number of female patients (*n* = 5). The third limitation is that the ameliorating effects of nGVS on body balance in the present study were observed under laboratory conditions. Further studies are needed to investigate the effect of nGVS on postural control, fall prevention, and quality of life in BV patients.

## Conclusions

In conclusion, we have shown that nGVS can induce a long-term post-stimulation effect on the improvement of postural stability for BV patients with a reduction in the high-frequency components of their postural movement. This ameliorating effect supports the potential of nGVS as a treatment for persistent unsteadiness in BV patients.

## Author contributions

CF and SI conceived of the study, conducted the experiments and wrote the manuscript and edited the manuscript for content. NE conducted the experiments and edited the manuscript for content. TK and YU provided statistical advice, performed statistical analysis and edited the manuscript for content. YY and TY conceived of the study, supervised interpretation of data and edited the manuscript for content.

### Conflict of interest statement

The authors declare that the research was conducted in the absence of any commercial or financial relationships that could be construed as a potential conflict of interest.
