# Supplementary material for: Noisy Galvanic Vestibular Stimulation Sustainably Improves Posture in Bilateral Vestibulopathy
Source: Front Neurol. 2018 Oct 22;9:900. doi: 10.3389/fneur.2018.00900 (PMC6204397; doi:10.3389/fneur.2018.00900)
Supplement: Supplementary file 1 [file Data_Sheet_1.docx]

***Supplementary Material***

**Noisy galvanic vestibular stimulation sustainably improves posture in bilateral vestibulopathy**

**Chisato Fujimoto, Naoya Egami, Takuya Kawahara, Yukari Uemura, Yoshiharu Yamamoto, Tatsuya Yamasoba, Shinichi Iwasaki^*^**

***Correspondence:**

Shinichi Iwasaki

E-mail: iwashin-tky@umin.ac.jp

**1 Supplementary Figures**

**Supplementary Figure 1 Change in NR of the velocity for all the 13 patients. (A)** Changes in the NR of the velocity for all the 13 patients in Session 1 are shown. **(B)** Changes in the NR of the velocity for all the 13 patients in Session 2 are shown.

NR = normalized ratio, ST = stimulation period, PST = post-stimulation period.

**Supplementary Figure 2 Change in NR of the area for all the 13 patients. (A)** Changes in the NR of the area for all the 13 patients in Session 1 are shown. **(B)** Changes in the NR of the area for all the 13 patients in Session 2 are shown.

NR = normalized ratio, ST = stimulation period, PST = post-stimulation period.

**Supplementary Figure 3 Change in NR of the RMS for all the 13 patients. (A)** Changes in the NR of the RMS for all the 13 patients in Session 1 are shown. **(B)** Changes in the NR of the RMS for all the 13 patients in Session 2 are shown.

NR = normalized ratio, RMS = root mean square, ST = stimulation period, PST = post-stimulation period.

**Supplementary Figure 4 NRs of the velocity, area, and RMS in Session 2.** Mean NRs of the velocity **(A)**, area **(B)**, and RMS **(C)** in Session 1 are shown. Dashed line indicates NR = 1.0.

NR = normalized ratio, RMS = root mean square, ST = stimulation period, PST = post-stimulation period. ^§§^P < 0.01, ^§§§^P < 0.001 (for contrast test). *P < 0.05 (for Student’s t-test).

**Supplementary Figure 5 MF calculated by power spectral analysis in Session 2.** MF in the AP axis (**A**) and the ML axis (**B**) in Session 1 are shown. Dashed line indicates mean MF at baseline.

AP = anterior-posterior, ML = medial-lateral, MF = mean frequency, ST = stimulation period, PST = post-stimulation period. ^§^P < 0.05 (for contrast test). *P < 0.05 (for Student’s t-test).

**Supplementary Figure 6 Score of subjective improvement in Session 2.** Mean score of the subjective improvement in Session 2 are shown.

ST = stimulation period, PST = post-stimulation period. ^§^P < 0.05 (for contrast test).*P < 0.05 (for Student’s t-test).
